# Examining the Structure and Utility of the EASE: Environmental Audit Scoring Evaluation Tool

**DOI:** 10.1093/geroni/igaf122.620

**Published:** 2025-12-31

**Authors:** Margaret Calkins, Migette Kaup

## Abstract

The designed environment is especially important for individuals with Alzheimer’s Disease and related dementias, but validated tools to assess physical environments lag behind theory and practice and omit best design practices. The Environmental Audit Scoring Evaluation tool (EASE) is a unique assessment tool for long-term care settings that is: 1) evidence-based, 2) dementia-inclusive (for both segregated and integrated living areas), and 3) specifically inclusive of household model settings. Founded on the principles of person-centered care practices, the tool specifically targets environmental characteristics that distinguish the values of residential living over institutional routines. The tool was administered in 228 living areas across US and Canada, including nursing homes, assisted living and memory care. The first paper addresses the analysis of the factorial structure of the EASE tool. The second paper examines environmental characteristics that differentiate between traditional versus person-centered care settings. The third paper explores the relationship between EASE evaluations and resident function as assessed with the MDS, identifying associations between environmental characteristics and outcomes of interest. The final paper explicates the use of the EASE as a tool to inform the process of change in seven traditional care settings seeking to become more person-centered.

